# Predatory attack on a bearded capuchin monkey by a *Boa constrictor*

**DOI:** 10.1007/s10329-025-01191-7

**Published:** 2025-04-15

**Authors:** Tatiane Valença, Emiliane Cardoso, Tiago Falótico

**Affiliations:** 1 Neotropical Primates Research Group, Ubajara Field Station, R. Jaime Lima, 20, Frecheirinha, São Paulo, CE 62340-000 Brazil; 2 University of São Paulo, São Paulo, Brazil; 3 Department for the Ecology of Animal Societies, Max Planck Institute of Animal Behavior, Constance, Germany; 4 Technological Primate Research Group, Max Planck Institute for Evolutionary Anthropology, Leipzig, Germany

**Keywords:** Predation risk, Prey–predator interaction, Alarm call, Boid snake, *Sapajus libidinosus*

## Abstract

Predation influences foraging strategies, as primates must avoid being preyed on while feeding. Some populations of capuchin monkeys are the only neotropical primates that customarily use the ground for nut-cracking. This time-consuming and focus-demanding foraging activity may increase susceptibility to predation events. However, there is currently no data on predation in tool-using *Sapajus* populations. We report a snake’s (*Boa constrictor*) predatory attack on a bearded capuchin monkey (*S. libidinosus*) in a tool-using population, as well as the other monkeys’ alarm calling and threat displays. We confirm these monkeys’ predation vulnerability and discuss how they balance foraging requirements with predation risk.

**Supplementary Information:**

The online version contains supplementary material available at 10.1007/s10329-025-01191-7.

## Introduction

Predation risk is a strong selective force that shapes animal morphology and behavior, influencing decision-making on foraging strategies (Lima and Dill 1990). Foraging is necessary to meet nutrient requirements while counterbalancing exposure to the risk of being attacked by a predator while feeding (Miller 2002). Although predation risk occupies a central discussion in foraging studies, in primates, it is usually indirectly presumed from risk perception behaviors (Campos and Fedigan 2014). Predatory attacks on primates are relatively rare events that are often difficult to observe and report (Libório and Moura Martins 2013).

Platyrrhine primates are vulnerable to predation by constrictors and venomous snakes (See reviews in Henderson 2023; Jack et al. 2020; Libório and Moura Martins 2013). Isbell (2006) suggests that platyrrhines are more arboreal than catarrhine primates due to their reduced ability to detect snakes camouflaged on the ground. Among platyrrhines, robust capuchins (genus *Sapajus*) present some unusual characteristics, with some savannah populations showing increased terrestriality (Falótico and Ottoni 2023), especially those using stone tools to crack nuts on the ground (Falótico 2022). In this situation, venomous and constrictor snakes may constitute a danger, although those monkeys regularly prey upon non-dangerous snakes (Falótico et al. 2018). Ottoni and Izar (2008) proposed that intensive stone tool use occurs in populations with reduced predation risk, but this argument is based on the risk perception behaviors of a few populations. There is currently a lack of data on predation in *Sapajus*, specifically from tool-using populations, and indeed no evidence that being on terrestrial tool sites is particularly dangerous for them.

Constrictor snakes, such as boas, are known to prey on platyrrhines ranging from small-sized species, like marmosets (Teixeira et al. 2016), to larger ones, such as howler monkeys (Quintino and Bicca-Marques 2013). In some cases, group members attacked the constrictor and rescued the victim (e.g., Tello et al. 2002). Although constricting snakes are considered to be potential predators of capuchins (Fragaszy et al. 2004), there is no direct evidence of them killing and eating *Sapajus*. In gracile capuchins (genus *Cebus*) there are two reports of predation attempts (Jack et al. 2020; Perry et al. 2003) and one of successful predation (Chapman 1986), indicating that they are vulnerable to constrictors. During the two attempts, group members—mainly the alpha male and female kin of the victims—mobbed and physically attacked the boa, and successfully rescued the juvenile victims. Although predation on *Sapajus* by constrictor snakes has not yet been reported, they exhibit alarm calls and mobbing behaviors upon detecting them (Falótico et al. 2018).

Here, we report a case of a bearded capuchin monkey (*S. libidinosus*) from a tool-use population killed by a *Boa constrictor*. We describe the environmental context in which the event occurred, the monkey victim, and the reactions of another capuchin group that approached the area. We also describe other interactions of the study population with snakes, including threats directed toward a boa detected at the side of a nut-cracking site.

## Methods

From 17 October to 18 December 2024, we followed a group of bearded capuchin monkeys (*S. libidinosus*), the Sertão Group, at the Ubajara National Park (Ceará, Brazil), totaling 242.6 h of contact time. This site is within Caatinga biome, but differs from typical savannah-like environment due to higher humidity. The park has two distinct regions, a highland area and a lowland area, the latter characterized by higher temperatures and lower humidity. This population has been studied since 2020 and is known to use stones as hammers and anvils to crack open macaúba (*Acrocomia aculeata*) and babaçu nuts (*Attalea speciosa*) mostly on the ground (Falótico et al. 2024a, 2024b). These monkeys also use stones and sticks as tools to access underground food, such as underground storage organs and trapdoor spiders (Valença et al. 2024). Sertão Group lives in the lowland area and has been studied since October 2021. We used the all-occurrences sampling (Altmann 1974) to record interactions with snakes during this period and ad libitum sampling (Altmann 1974) to record behaviors of neighboring groups, including the predation event reported here.

## Results

### Predatory attack on a capuchin monkey

**Fig. 1:**
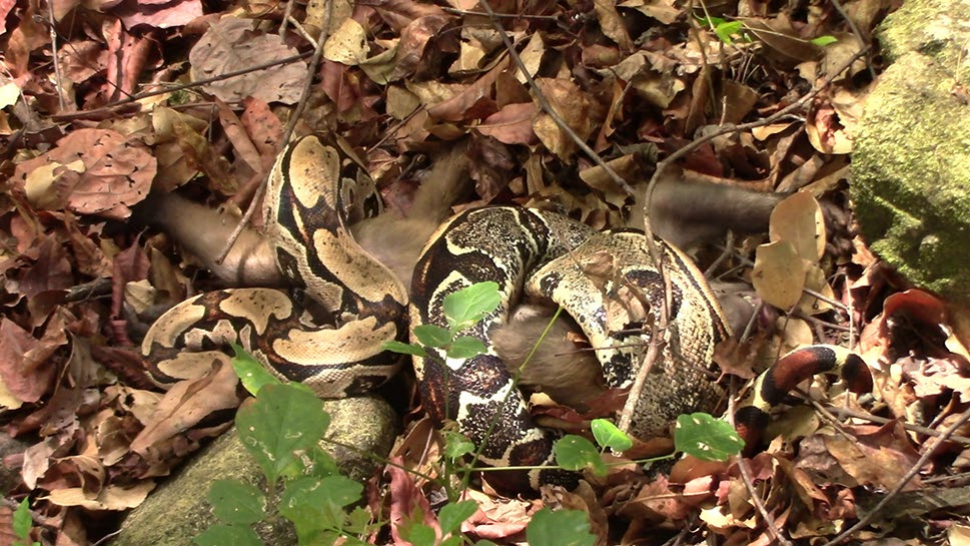
*Boa constrictor* coiled around a juvenile male bearded capuchin monkey (*Sapajus libidinosu*s) at Ubajara National Park, Brazil. Frame from a video by E.C

On 11 December 2024, E.C. was following the Sertão Group since 08:12. It was the end of the dry season. At 12:10, she heard the monkeys from the studied group emitting low-intensity alarm calls and approached the area. She then saw a *Boa constrictor* approximately 1.5 m long, on the ground, coiled around a bearded capuchin monkey from another group (Fig. 1, Video S1). The monkey appeared dead, emitting no vocalizations and showing no signs of breathing or moving. The event occurred in an area with open vegetation (3°49ʹ39.540″ S 40°53ʹ34.620″ W), with sparse trees and few bushes. The animals—the snake and the victim—were on the ground among the light-brownish leaf litter (similar in color to the snake), close to stones, and near a mango tree; some mango fruits with monkey bite marks lay close to them on the ground. About 7 m away, there was a nut-cracking site with remains of macaúba nuts.

The Sertão Group monkeys were foraging on a mango tree close to the area. At least two adult females and the alpha male moved closer to the snake and victim while remaining in the trees (approximately 2.5 m), looked intensely at the snake and alarm-called, while the alpha male also threatened the snake from up in the trees (Video S1). The alarm calls and threats appeared to be of low intensity—brief and not very loud—compared to the reactions to some snakes that we have witnessed in previous years. The Sertão Group did not mob the snake, and no member approached it to within touching distance. The victim’s group is unknown and no other capuchins were observed by E.C. in the moment she found the corpse.

The snake remained coiled around the capuchin, occasionally moving its head slightly. At 13:01, the snake started to move its head around the corpse, uncoiled, pulled the monkey a few cm and positioned its mouth around the monkey’s head, apparently starting to swallow it (Video S1). When T.V. approached the snake to get more information about the capuchin, the snake—apparently disturbed—stopped swallowing and expelled it from its mouth. T.V., therefore, moved away, looking from a distance to the snake to see whether the snake would resume swallowing the monkey. The snake instead moved away and hid under the leaf litter, close to the stones, disappearing from view. T.V. waited 15 min and, as the snake did not return, she approached and collected the corpse, inspected it for 10 min, and then returned it to the same place before leaving the area to avoid causing further disturbance. No reaction from the snake was observed.

The corpse was that of an older juvenile male (Fig. S1), almost the size of an adult female but without grown tufts, and estimated to be around 4.5 years old. There were snake bite marks on the inner part of the right thigh (Fig. S2). The individual appeared generally healthy and well nourished, with intact fingers and toes and no visible ectoparasites. Some fluid was visible at the left nostril, along with a tiny wound to the side of the left eye; and a small area on the tail was hairless. Almost all teeth were present and intact, although the upper canines showed evidence of a kind of linear enamel hypoplasia (Fig. S3, Chollet and Teaford 2010).

The following day (12 December), at 09:10, T.V. returned to the site, and found neither corpse nor snake. There was no carcass smell and no vultures or other carcass-consuming scavengers. The snake might have returned and ingested the monkey, but we cannot rule out the possibility that another species may have consumed or taken it away.

### Other capuchin–snake interactions

**Fig. 2:**
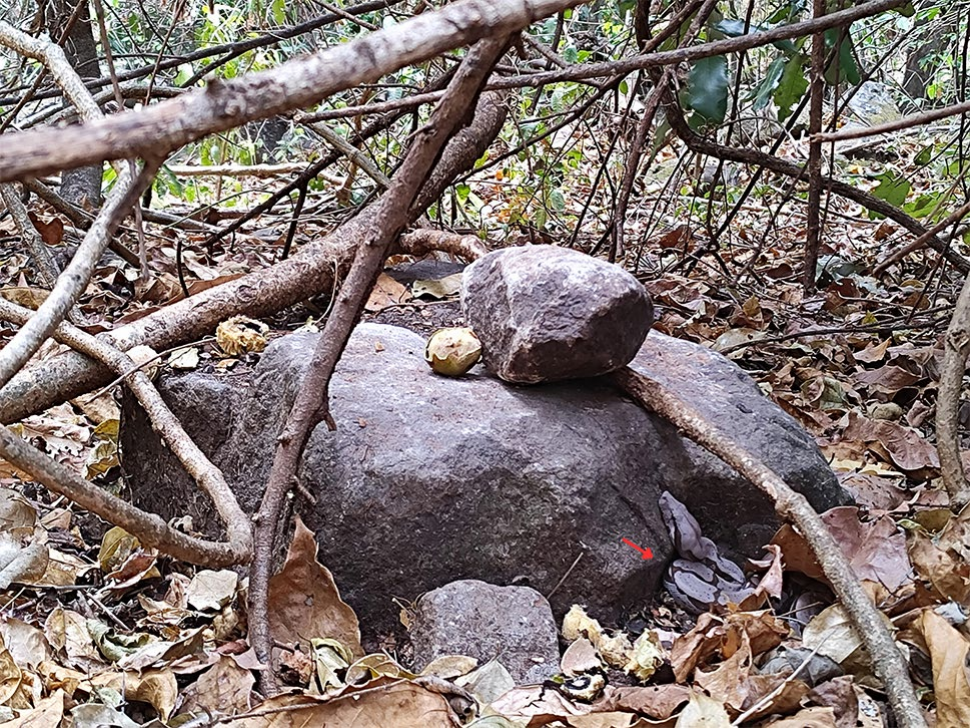
*Boa constrictor* observed at a recently used macaúba nut-cracking site. The picture was taken on 28 October 2024, at 10:18 while following the Sertão group. Capuchin monkeys alarm called and threatened this snake. Photo by T.V

We observed four cases of monkeys from the focal group threatening snakes through alarm calls, facial expressions, branch shaking, and branch-dropping. Three of them involved chicken snakes (*Spilotes pullatus*) that were up in the trees and did not pose a threat to them. The last one involved a *Boa constrictor* on the ground, at a nut-cracking site (Fig. 2). The alpha male and one adult female threatened it. Two adult males and another adult female approached and looked intently at the area where the individuals were threatening the boa, but seemed not to gaze at the exact place it was. Many juveniles and infants alarm called for a prolonged period. The snake remained in the same spot, while the capuchins eventually moved away. We also recorded an adult female capturing and eating a small snake; a juvenile who observed her (Video S2) later recovered the discarded remains and consumed them.

## Discussion

To our knowledge, this is the first report of a predatory attack by a snake on robust capuchin monkeys (genus *Sapajus*) and the first report of a predatory attack on a capuchin monkey from a tool-use population. This report confirms these monkeys’ vulnerability to snake predation.

The predatory event was similar to other cases observed in *Cebus* (Jack et al. 2020; Perry et al. 2003; Chapman 1986), with the prey being a juvenile, suggesting relatively higher vulnerability of this age category to snakes. This vulnerability might not be because of their smaller size alone but because of their underdeveloped anti-predator detection and avoidance skills, which seem to sharpen during later stages of ontogeny (Meno et al. 2013).

In two of three predation reports involving *Cebus*, two or more group members mobbed, hit and bit the snake, and successfully rescued the victims, showing these monkeys’ ability to counter predators collectively (Jack et al. 2020; Perry et al. 2003). In the present case, other monkeys looked at and threatened the snake, and alarm called, but these behaviors did not appear intense, and no mobbing occurred. The lack of a more intense reaction may be explained by the victim belonging to another group, and those individuals who did not react were avoiding unnecessarily risky exposure. Or it could be explained by the fact that the group did not observe the snake attacking. Alternatively, it seems possible that the monkeys perceived that the victim was already dead, or at least silent and inert. However, monkeys’ and other primates’ comprehension of death and its consequences is unclear (e.g., Valença and Falótico 2023).

The snake and the capuchin were found on the ground and the monkey had bites on the right thigh. This suggests that the snake might have caught the monkey from below, possibly on the ground, which is in line with the suggestion that platyrrhines are more vulnerable on the ground (Miller 2002), particularly due to the lower ability to detect snakes (Isbell 2006). However, we cannot discard the possibility that the catch occurred in the trees, after which both fell to the ground. Boa constrictors are known to catch primates both on the ground and in the trees (Henderson 2023).

The closest nut-cracking site was 7 m from the area. Although snakes are able to drag their prey, this suggests that the victim was not caught during nut-cracking activities. However, our observation of group alarm calling and threatening a boa at a nut-cracking site, along with the confirmed vulnerability of *Sapajus* to boas, increases the possibility of attacks occurring during monkey’s nut-cracking. Moreover, since 2021 in Ubajara National Park, we have seen two other snakes right beside capuchin tool sites, one boa and one coral-like snake (T.V., pers. obs.). These sightings support the view that capuchin monkeys are vulnerable to snakes at tool sites (Visalberghi et al. 2007). More long-term studies on *Sapajus* tool-using populations are required to test the suggestion that they have a relatively low predation risk (Ottoni and Izar 2008) and that tool-use may increase the risk of attack on the ground, including comparisons of vulnerability to predation with other terrestrial activities.

We observed a snake being caught and consumed by capuchins. *Sapajus* are the only primates besides humans known to regularly eat non-dangerous snakes (Falótico et al. 2018). In our observation, a juvenile consumed the remains of a snake captured and mostly eaten by an adult. Given their vulnerability to *B. constrictor*, capuchins’ abilities to distinguish among snakes and take defensive behaviors are important for foraging with at least some protection against predation. Studies on the ontogeny of anti-predatory behaviors, as well as comparisons of sensory systems of *Sapajus* and *Cebus*, may help to elucidate how these primates acquire the ability to differentiate prey from predator snakes.

In conclusion, *Sapajus* are vulnerable to snakes, including this population that uses tools and preys on snakes. Tool use to crack palm nuts demands a lot of attention and can potentially decrease an individual’s vigilance (Barrett et al. 2018), increasing the risk of predatory attacks. Conversely, palm nuts kernels are rich in lipids, and their consumption significantly increases capuchin monkeys’ net energy gain (Izar et al. 2022). Similarly, snakes are a potentially rich source of protein, but consuming or attempting to consume the wrong kind of snake can be fatal (Pissinatti et al. 2017). Exploring how *Sapajus* counterbalances foraging requirements and exposure to risk can advance our general understanding of the evolution of primate foraging strategies in complex three-dimensional landscapes.

## Supplementary Information

Below is the link to the electronic supplementary material.

Supplementary file1 (DOCX 1906 KB)

Supplementary file2 (MP4 66289 KB)

Supplementary file3 (MP4 27897 KB)

## Data Availability

All data supporting the findings of this study are included within this paper and its Supplementary Information. The complete video recording is available from the corresponding author upon request.

## References

[CR1] Altmann J (1974) Observational study of behavior: sampling methods. Behaviour 49:227–266. 10.1163/156853974x00534 4597405 10.1163/156853974x00534

[CR2] Barrett BJ, Monteza-Moreno CM, Dogandžić T, Zwyns N, Ibáñez A, Crofoot MC (2018) Habitual stone-tool-aided extractive foraging in white-faced capuchins, *Cebus Capucinus.* R Soc Open Sci 5:181002. 10.1098/rsos.181002 30225086 10.1098/rsos.181002 PMC6124021

[CR3] Campos FA, Fedigan LM (2014) Spatial ecology of perceived predation risk and vigilance behavior in white-faced capuchins. Behav Ecol 25:477–486. 10.1093/beheco/aru005

[CR4] Chapman CA (1986) *Boa constrictor* predation and group response in white-faced cebus monkeys. Biotropica 18:171–172

[CR5] Chollet MB, Teaford MF (2010) Ecological stress and linear enamel hypoplasia in *Cebus*. Am J Phys Anthropol 142:1–6. 10.1002/ajpa.21182 19918987 10.1002/ajpa.21182

[CR6] Falótico T (2022) Robust capuchin tool use cognition in the wild. Curr Opin in Behav Sci 46:101170. 10.1016/j.cobeha.2022.101170

[CR7] Falótico T, Ottoni EB (2023) Greater tool use diversity is associated with increased terrestriality in wild capuchin monkeys. Am J Biol Anthropol 181:312–317. 10.1002/ajpa.24740 37067352 10.1002/ajpa.24740

[CR8] Falótico T, Verderane MP, Mendonca-Furtado O, Spagnoletti N, Ottoni EB, Visalberghi E, Izar P (2018) Food or threat? Wild capuchin monkeys (*Sapajus libidinosus*) as both predators and prey of snakes. Primates 59:99–106. 10.1007/s10329-017-0622-4 28918605 10.1007/s10329-017-0631-x

[CR9] Falótico T, Valença T, Verderane MP, Santana BC, Sirianni G (2024a) Mapping nut-cracking in a new population of wild capuchin monkeys (*Sapajus libidinosus*) at Ubajara National Park. Brazil Am J Primatol 86:e23595. 10.1002/ajp.23595 38224002 10.1002/ajp.23595

[CR10] Falótico T, Macedo AC, de Jesus MA, Espinola T, Valença T (2024b) Nut-cracking success and efficiency in two wild capuchin monkey populations. R Soc Open Sci 11:240161. 10.1098/rsos.240161 39092146 10.1098/rsos.240161 PMC11293797

[CR11] Fragaszy DM, Visalberghi E, Fedigan LM (2004) The complete capuchin: the biology of the genus Cebus. Cambridge University Press

[CR12] Henderson RW (2023) A review of predation by *Boa constrictor* (Squamata: Boidae): what, when, and where. Herpetol Conserv Biol 18:569–578

[CR13] Isbell LA (2006) Snakes as agents of evolutionary change in primate brains. J Hum Evol 51:1–35. 10.1016/j.jhevol.2005.12.012 16545427 10.1016/j.jhevol.2005.12.012

[CR14] Izar P, Peternelli-dos-Santos L, Rothman JM, Raubenheimer D, Presotto A, Gort G, Visalberghi E, Fragaszy DM (2022) Stone tools improve diet quality in wild monkeys. Curr Biol 32:4088–4092. 10.1016/j.cub.2022.07.056 35985326 10.1016/j.cub.2022.07.056

[CR15] Jack KM, Brown MR, Buehler MS, Cheves Hernadez S, Ferrero Marín N, Kulick NK, Lieber SE (2020) Cooperative rescue of a juvenile capuchin (*Cebus imitator*) from a *Boa constrictor*. Sci Rep 10:16814. 10.1038/s41598-020-73476-4 33033278 10.1038/s41598-020-73476-4 PMC7544904

[CR16] Libório RA, Moura Martins M (2013) Body size in predator–prey interactions: an investigation of Neotropical primates and their predators. Stud Neotrop Fauna Environ 48:81–87. 10.1080/01650521.2013.789724

[CR17] Lima SL, Dill LM (1990) Behavioral decisions made under the risk of predation: a review and prospectus. Can J Zool 68:619–640. 10.1139/z90-092

[CR18] Meno W, Coss RG, Perry S (2013) Development of snake-directed antipredator behavior by wild white-faced capuchin monkeys: I Snake-Species Discrimination. Am J Primatol 75:281–291. 10.1002/ajp.22106 23229464 10.1002/ajp.22106

[CR19] Miller LE (2002) Eat or be eaten: Predator sensitive foraging among primates. Cambridge University Press, Cambridge, 297

[CR20] Ottoni EB, Izar P (2008) Capuchin monkey tool use: overview and implications. Evol Anthropol 17:171–178. 10.1002/evan.20185

[CR21] Perry S, Manson JH, Dower G, Wikberg E (2003) White-faced capuchins cooperate to rescue a groupmate from a *Boa constrictor*. Folia Primatol 74:109–101. 10.1159/000070008 10.1159/000070008 12778926

[CR22] Pissinatti A, Chagas WN, da Cruz JB, de Araújo ST, Beck BB (2017) Snake incident as a limiting factor in the process of reintroduction of lion tamarins to their habitat. *Leontopithecus* Lesson, 1840 (Callitrichidae-Primates). In: Ferreira RG (ed) A Primatologia no Brasil, vol 14. Editora UFPE, pp 212–225

[CR23] Quintino EP, Bicca-Marques JC (2013) Predation of *Alouatta puruensis* by *Boa constrictor*. Primates 54:325–330. 10.1007/s10329-013-0377-z 23917944 10.1007/s10329-013-0377-z

[CR24] Teixeira DS, Dos Santos E, Leal SG, de Jesus AK, Vargas WP, Dutra I, Barros M (2016) Fatal attack on black-tufted-ear marmosets (*Callithrix penicillata*) by a *Boa constrictor*: a simultaneous assault on two juvenile monkeys. Primates 57:123–127. 10.1007/s10329-015-0495-x 26467338 10.1007/s10329-015-0495-x

[CR25] Tello NS, Huck M, Heymann EW (2002) *Boa constrictor* attack and successful group defence in moustached tamarins, *Saguinus mystax*. Folia Primatol 73:146–148. 10.1159/000064795 10.1159/000064795 12207064

[CR26] Valença T, Falótico T (2023) Life and death of a disabled wild capuchin monkey infant. Primates 64:207–213. 10.1007/s10329-023-01052-1 36790569 10.1007/s10329-023-01052-1

[CR27] Valença T, Affonço GO, Falótico T (2024) Wild capuchin monkeys use stones and sticks to access underground food. Sci Rep 14:10415. 10.1038/s41598-024-61243-8 38710945 10.1038/s41598-024-61243-8 PMC11074112

[CR28] Visalberghi E, Fragaszy D, Ottoni E, Izar P, de Oliveira MG, Andrade FRD (2007) Characteristics of hammer stones and anvils used by wild bearded capuchin monkeys (*Cebus libidinosus*) to crack open palm nuts. Am J Phys Anthropol 132:426–444. 10.1002/ajpa.20546 17177182 10.1002/ajpa.20546

