# Supplementary material for: Predatory attack on a bearded capuchin monkey by a Boa constrictor
Source: Primates. 2025 Apr 15;66(4):349–53. doi: 10.1007/s10329-025-01191-7 (PMC12202570; doi:10.1007/s10329-025-01191-7)
Supplement: Supplementary file 1 — Supplementary file1 (DOCX 1906 KB) [file 10329_2025_1191_MOESM1_ESM.docx]

**Supplementary material**


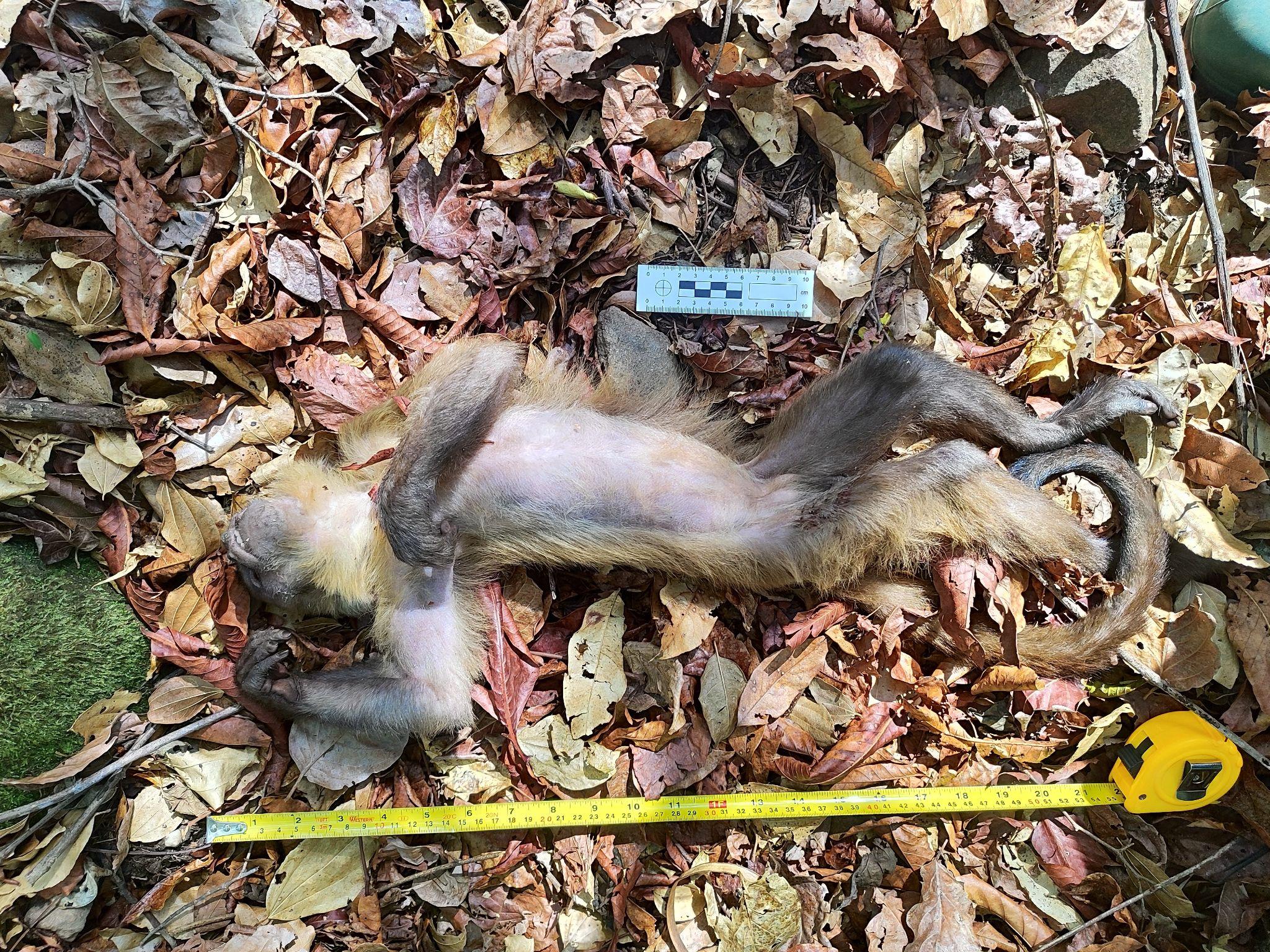


**Fig. S1** The corpse of the bearded capuchin monkey attacked by *Boa constrictor*. Ubajara National Park, Brazil.


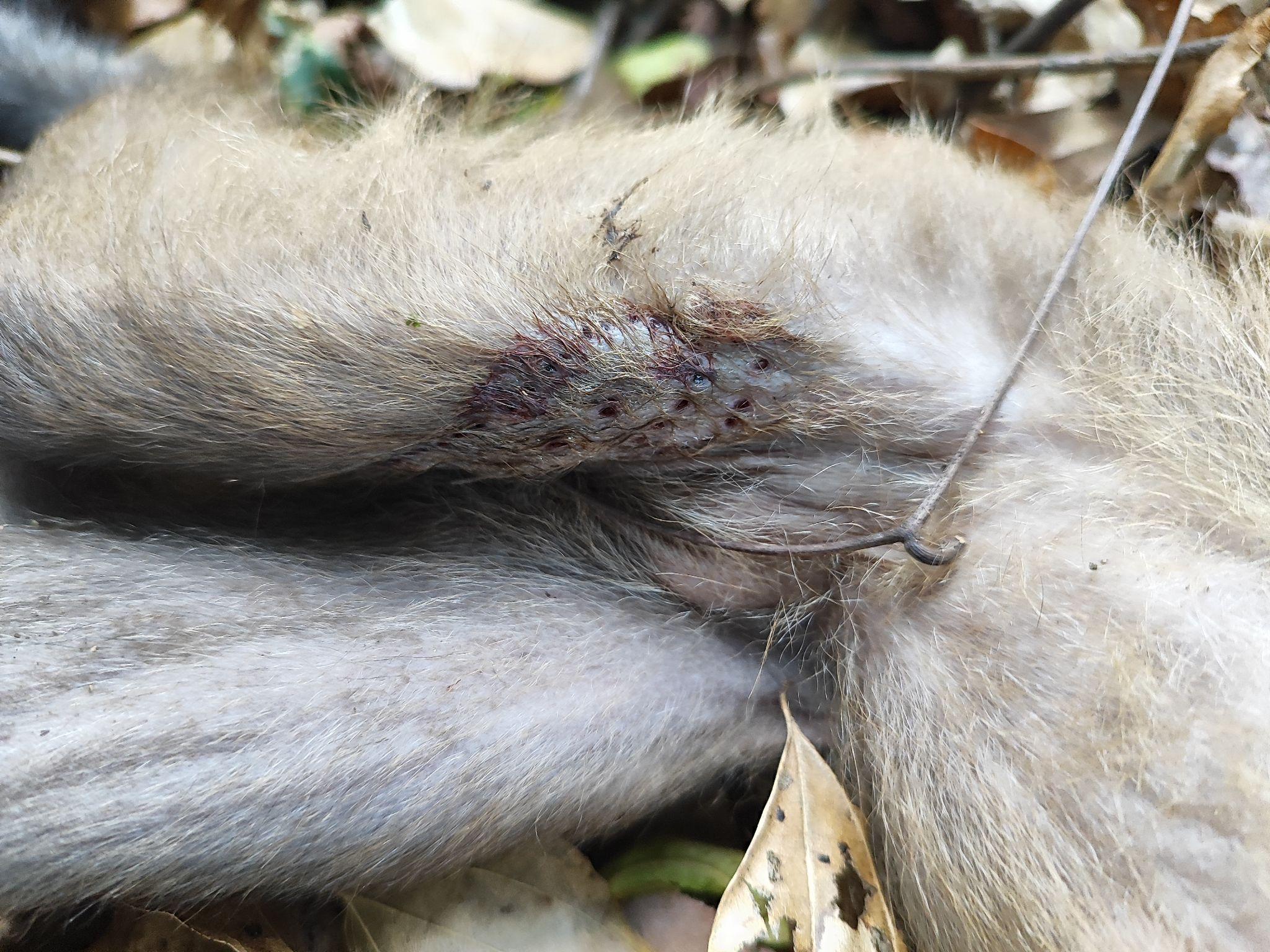


**Fig. S2** Bite marks in the right thigh of the bearded capuchin monkey.


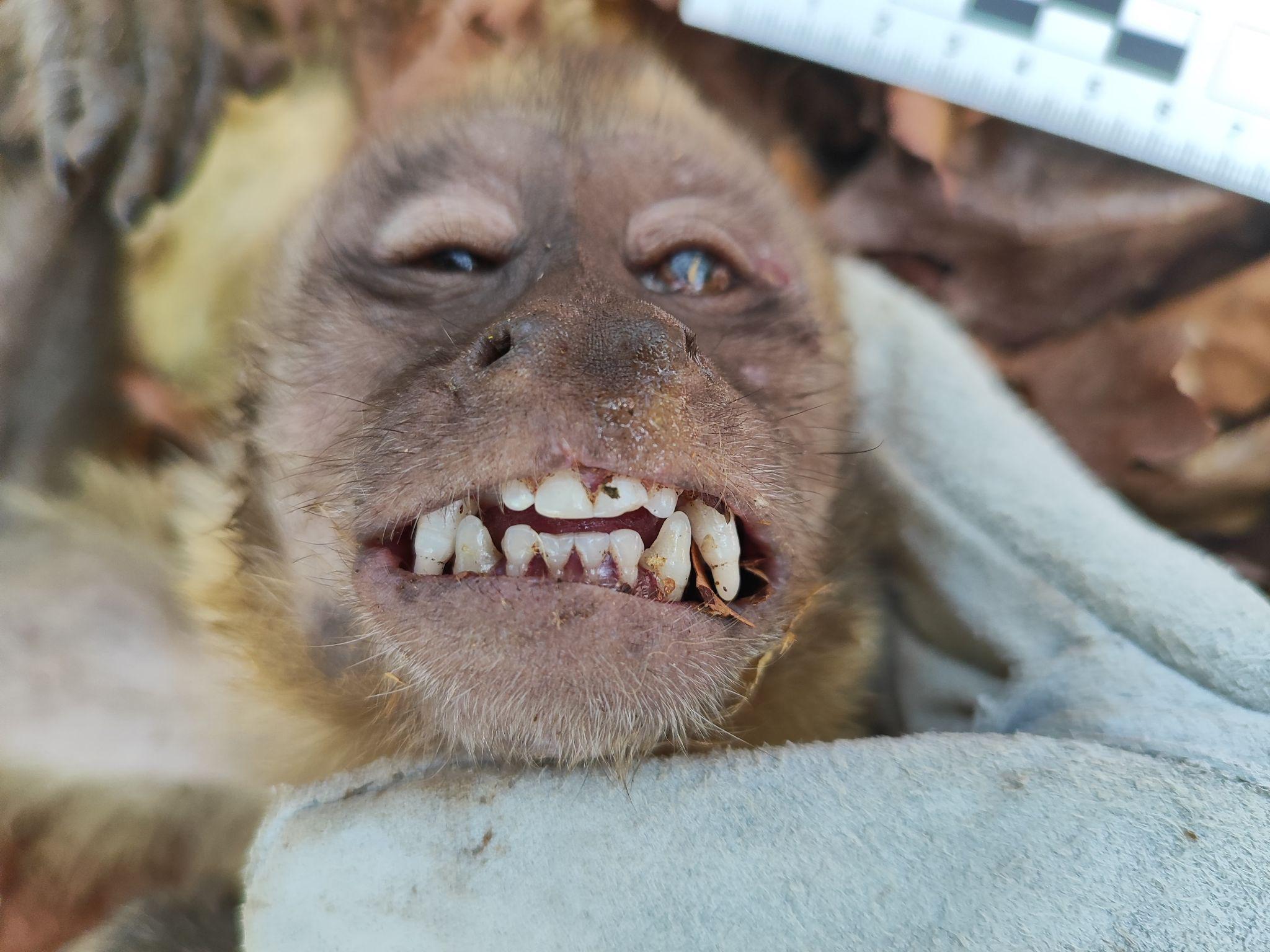


**Fig. S3** Superior canines seemed to present a kind of linear enamel hypoplasia

**Subtitles of videos**

**Video S1** *Boa constrictor* coiled around a capuchin monkey, alpha male threatening the snake, and snake positioning its mouth around the monkey’s head.

**Video S2** Female capuchin monkey preying on a snake while a juvenile observes and eats the remains.
